# Evaluating the cost-effectiveness of biologic treatments for psoriatic arthritis: can we make better use of patient data registries?

**DOI:** 10.1007/s10067-017-3703-9

**Published:** 2017-06-13

**Authors:** Thomas Patton, Laura Bojke, Matthew Walton, Andrea Manca, Philip Helliwell

**Affiliations:** 1 Centre for Health Economics, University of York, York, YO10 5DD UK; 2 Centre for Reviews and Dissemination, University of York, York, YO10 5DD UK; 3 Faculty of Medicine and Health, University of Leeds, Leeds, LS2 9JT UK

**Keywords:** Biologics, Cost-effectiveness, Decision model, Psoriatic arthritis, Registry data

## Abstract

The primary aim of this study is to explore the extent to which registry data may fulfill the evidence requirements of cost-effectiveness analysis (CEA) studies evaluating biologic therapies for the treatment of psoriatic arthritis (PsA), where trial data are lacking or insufficient. In addition, the paper aims to identify how future data collection in PsA registries might be better tailored to inform CEA research. A review of the literature was performed to identify existing registries containing PsA patients. Where possible, information was extracted on the design and characteristics of the registries. The registries were then appraised according to a set of criteria that was formulated based on the methods currently used to model PsA in the CEA literature. A review of the literature identified 21 potentially relevant registries from around the world containing patients with PsA. There was substantial variation regarding the extent to which the registries, as a whole, were useful for the purposes of CEA studies. There were also notable disparities found in terms of the accessibility of the registries to researchers. The critical review conducted in this study showed that all of the registries identified are potentially useful, at least in some degree, for the purposes of informing CEA studies in PsA. However, no individual registry on its own was found to meet all of the evidence requirements when considering how the disease has been modeled previously.

## Introduction

In recent years, an increasing number of biologic therapies have been made available for the treatment of psoriatic arthritis (PsA). Biologics are particularly effective at controlling the symptoms of PsA and have been shown to delay disease progression in terms of joint erosion [1]. However, these treatments are expensive, and within resource constrained systems, their value for money has been assessed by health technology assessment (HTA) agencies to determine whether they should be approved for reimbursement in public health care systems [2–4]. Many HTA agencies require robust evidence demonstrating that a drug therapy is cost-effective, as well as clinically effective, to receive a positive reimbursement decision [5]. As a result, cost-effectiveness evidence has come to play a prominent role in decisions regarding the approval of biologic treatments in many settings. Unfortunately, the development of robust cost-effectiveness evidence for PsA treatments has often been hindered by deficiencies in the evidence base. The short-term nature of many phase 3 trials in this area means that assumptions regarding the long-term efficacy of biologics are required to investigate the cost-effectiveness of biologic therapies over the remaining lifetime of an average patient. Consequently, this can impose additional uncertainty surrounding the results and, ultimately, reduce confidence in a decision to accept or reject a treatment for reimbursement.

Whilst HTA bodies may have appraised many of the biologic drugs available, albeit with suboptimal evidence, they have faced a greater challenge in establishing an optimum treatment sequence. Research has shown that switching between biologic therapies should be considered in patients experiencing treatment failure either due to primary non-response, secondary loss of efficacy or adverse events [6]. Unfortunately, the scope for evaluating alternative treatment sequences in the context of a clinical trial is limited, given the need to capture switches between multiple different treatments, necessitating a longer follow-up period.

Real-world data collected for purposes of research, in particular registry data, has the potential to circumvent many of the aforementioned issues associated with the use of trial evidence to inform CEA studies of biologic drugs in PsA patients. There are multiple registries, such as the British Society for Rheumatology Biologics Registry (BSRBR) and a registry in Denmark (DANBIO), that capture information on PsA patients receiving a variety of treatments, including biologic and non-biologic therapies. One of the main advantages of using evidence from registries such as these is that they follow patients for up to 15 years and over multiple lines of treatment [7].

The aims of this paper are twofold. It first aims to explore the extent to which existing registry data can be used to inform CEA studies involving biologic therapies for the treatment of PsA. The second aim of the paper is to identify how data collection in PsA registries might be improved to inform future CEA research.

We start by identifying relevant literature pertaining to PsA registry data and use this to establish a list of previous or ongoing patient registries around the world. Next, we extract information about the design and characteristics of the each of the registries, including whether or not the registries employ measures of disease activity that are relevant from a clinical and economic perspective. The registries are then appraised according to a set of criteria that was formulated based on the methods currently used to model PsA in the CEA literature. Finally, the findings are used to inform recommendations regarding the use and collection of data in PsA patient registries for the purposes of CEA.

## Methods

### Identifying registries

Reflecting the anticipated difficulty in identifying registry studies, we have used a pearl growing approach [8] to generate a full list of registries containing PsA patients. The pearl in this instance is a review of clinical registries in psoriatic arthritis published in 2011 [7]. This is supplemented with focused internet searches to identify registries without any associated publications that may be reported in the gray literature (e.g. policy documents or websites). Furthermore, expert clinical opinion was sought to identify registries falling outside of both the published and gray literature.

For the purposes of this study, we define registry data according to the definition set out in a report commissioned by the Agency for Healthcare Research and Quality (AHRQ) on the development and evaluation of registry data on patient outcomes [9]. This report defined a registry as “an organized system that uses observational study methods to collect uniform data (clinical and other) to evaluate specified outcomes for a population defined by a particular disease, condition, or exposure, and that serves one or more predetermined scientific, clinical, or policy purposes”. Thus, this definition covers a broad range of study types, including registries for health care products (e.g. post-marketing surveillance studies and single-arm open-label trials), health services registries and disease or condition specific registries.

### Data extraction and critical appraisal

To determine which information might be useful from available registries, the following data was extracted, where available: population included in the registry (including PsA subgroups, duration of disease on entering database), number of patients, setting (country), treatments received, follow-up duration, timing of patient follow-up, patient numbers at each follow-up, outcomes recorded (including QoL and costs) and data availability (barriers to access, industry funding).

In addition, a series of questions were formulated (see Box 1). These were informed by challenges noted in previous applied papers and a recent consensus statement on cost-effectiveness modeling in RA and PsA. Specifically, Madan and colleagues defined six components that can be used to describe existing cost-effectiveness models: initial response, longer-term disease progression, mortality, quality-adjusted life year estimation, resource use and the selection and interpretation of data [10]. A subsequent study identified model components—including long-term disease progression, the duration of treatment effects, health care resource usage and mortality—where registry data could play an important role in resolving current deficiencies in the use of evidence [11]. We sought to identify available and appropriate registry data according to these components.

**Box 1:** Critical appraisal questions

Q1. Which outcomes, if any, are collected to determine whether or not an initial treatment response (12/16 week) is achieved? Q2. Which outcomes, if any, are collected to assess the effect of treatments on the disease symptoms? Q3. Was information on the timing of patient withdrawal collected? Moreover, was the reason for patient withdrawal recorded? Q4. Which outcomes, if any, are collected to assess long-term disease progression, in terms of arthritis-related progression? Q5. Were outcomes collected in way that would allow long-term disease progression to be estimated (i.e. beyond the initial treatment period)? Q6. Was the data collected in a way that would permit the estimated effect of treatment withdrawal on disease progression (e.g. estimate any potential rebound effect)? Q7. Does the registry data include information on health care resource use in patients? Alternatively, can the registry data be linked to external data on health care resource use in patients? Q8. Does the data permit analyses to model the relationship between disease severity and mortality risk? Q9. Does the data permit treatment response and treatment effectiveness to be determined at various stages of the treatment sequence (i.e. first-line treatment, second-line treatment etc.)?	

**Fig. 1:**
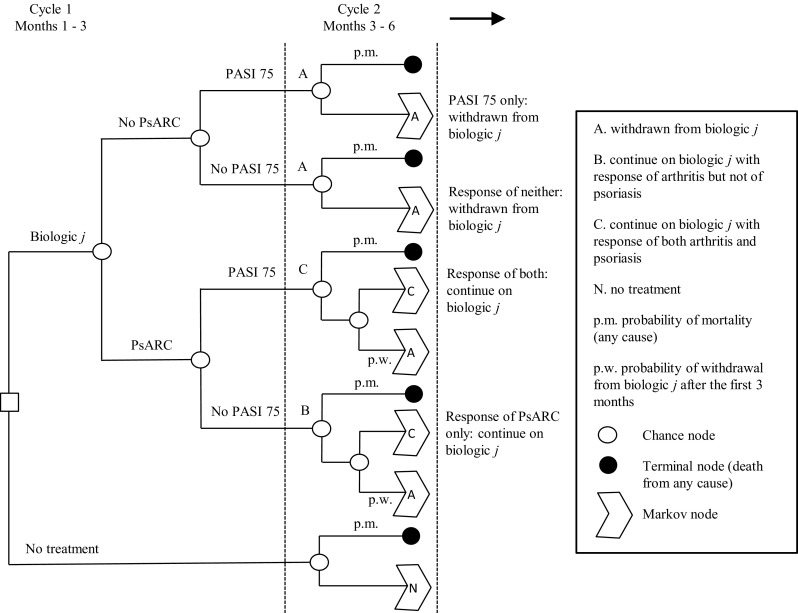
Model schematic for PsA. Figure reproduced with permission from Rodgers M, Epstein D, Bojke L et al. Etanercept, infliximab and adalimumab for the treatment of psoriatic arthritis: a systematic review and economic evaluation. Health Technol Assess 2011; 15: 1-329

In terms of outcomes that were collected in each of the registries, availability of data from each of the registries were reviewed in terms of their ability to inform a model structure similar to that recently developed as part of the appraisal process of the National Institute for Health and Care Excellence (NICE) [12]. This model structure, set out in Fig. 1, was assumed to reflect best current practice in the methods used to model PsA in the CEA literature. Here, initial response to treatment is determined by the PsA Response Criteria (PsARC) and the Psoriasis Area Severity Index (PASI). Subsequent disease activity is modeled using the Health Assessment Questionnaire (HAQ) and PASI, where HAQ is assumed to increase (worsen) without treatment, and PASI is assumed to remain constant.

## Results

### Registries containing PsA patients

Identified registries are shown in Table 1 below, along with the country in which they are founded. Twenty-one registries were identified in total, pertaining to 18 different countries. There was very little information that could be acquired for the Czech, Icelandic and Turkish registries, and as such, these registries are excluded from further discussions.

**Table 1:** Identified registries

	Full name	Country
ARAD	Australian Rheumatology Association Database	Australia
ARTIS	Antirheumatic Therapies in Sweden	Sweden
ATTRA	Czech National Registry	Czech Republic
BIOBADA-BRASIL	Brazilian Biologic Registry	Brazil
BIOBADA-SER 2.0	Spanish Registry for adverse events of biological therapies in rheumatic diseases	Spain
BSRBR	British Society of Rheumatologists Biologics Register	UK
CORRONA	Consortium of rheumatology researchers of north America	USA
DANBIO	Danish Database for Biological Therapies	Denmark
GISEA	Italian Group for the Study of Early Arthritis	Italy
HRBT	Hellenic Registry of Biologic Therapies	Greece
ICEBIO	–	Iceland
NOAR	Norfolk Arthritis Registry	UK
NOR-DMARD	Norwegian Anti-rheumatic Drug Register	Norway
PsART	Psoriatic Arthritis Registry of Turkey	Turkey
PsoBEST	German Psoriasis Registry PsoBest	Germany
Reuma	Rheumatic Diseases Portuguese Register	Portugal
ROB-FIN	National Register of Biological treatment in Finland	Finland
SCQM	Swiss Clinical Quality Management in Rheumatic Diseases	Switzerland
SSATG	South Swedish Arthritis Treatment Group	Sweden
SwePsA	Swedish Early Psoriatic Arthritis Registry	Sweden
UoT Psoriatic Arthritis	–	Canada

### Data extraction

Table 2 shows which data are available in each of the registries for the purposes of informing CEA studies in PsA patients. The large majority of registries do not capture any treatment response within 3 to 6 months of patients starting a new treatment, the time horizon typically specified in PsA clinical trials following BSR/BAD guidance [13, 14]. The Reuma and PsoBEST registries were the only ones found to collect evidence in keeping with the recommendations set out by Madan and colleagues [11], i.e. PsARC and PASI75 collected 3 months after the initiation of treatment. A further two registries collect PASI75 response either at 6 months or beyond (GISEA, SwePSA). The paucity of short-term data on treatment effects may reflect the intended nature of the registries, namely that they are not designed for the purposes of clinical research but also for operational reasons, as well as cost restrictions on the collection of data. A further complication regarding the estimation of treatment effects is that the patients may not enter the registry until they are established on treatment.

**Table 2:** Data available from registries

	Measure of treatment response at 3 months	Measure of disease progression at 3 months	Measure of disease progression beyond 3 months	Patient withdrawal from treatment			Health care resource use data	Mortality data	Multi-stage treatment sequence
				Date	Reason	Measure of disease progression			
ARAD	None	None	HAQ, EQ-5D, SF-36, AQoL	✓	✓	⨯	ARAD can be linked to Medicare Australia Data	ARAD can be linked to National Death Index	✓
ARTIS	None	HAQ	HAQ	✓	✓	Unclear	Identification number can be linked to the National Patient Register	Identification number can be linked to Census Data	✓
BIOBADA-BRASIL	None	None	None	✓	✓	⨯	Unclear	Unclear	Unclear
BIOBADA-SER 2.0	None	None	None	✓	✓	⨯	Unclear	Unclear	Unclear
BSRBR	None	None	HAQ, SF-36	✓	✓	Unclear	Unclear	Yes	✓
CORRONA	None	Unclear	HAQ, EQ-5D	Unclear	Unclear	Unclear	Unclear	Unclear	Unclear
DANBIO	None	None	HAQ	✓	✓	Unclear	Unclear	Identification number can be linked to National Death Registry	✓
GISEA	None	None	HAQ, EQ-5D	✓	✓	Unclear	Unclear	Yes	✓
HRBT	None	None	Modified HAQ for physical function	✓	✓	✓	Unclear	Unclear	✓
NOAR	None	None	HAQ, SF-36	Unclear	Unclear	Unclear	Unclear	Unclear	Unclear
NOR-DMARD	ACR	Modified HAQ, SF-36	Modified HAQ, SF-36	✓	✓	Unclear	Yes	Unclear	✓
PsoBEST	PASI75^a^	HAQ, EQ-5D	HAQ, EQ-5D	✓	Unclear	Unclear	Unclear	Yes	✓
Reuma	PsARC	HAQ, SF-36	HAQ, SF-36	✓	✓	Unclear	Unclear	Unclear	Unclear
ROB-FIN	ACR	HAQ	HAQ	✓	✓	Unclear	Unclear	Unclear	✓
SCQM	None	HAQ, SF-36, EQ-5D	HAQ, SF-36, EQ-5D	✓	✓	Unclear	Unclear	Unclear	Unclear
SSATG	None	HAQ, EQ-5D	HAQ, EQ-5D	✓	✓	Unclear	Unclear	Unclear	✓
SwePsA	None	None	HAQ, SF-36	Unclear	Unclear	Unclear	Unclear	Unclear	⨯
UoT Psoriatic Arthritis	None	None	HAQ, SF-36	✓	✓	Unclear	Unclear	Unclear	✓

^a^PASI score collected from which PASI75 can be calculated

The evidence pertaining to disease progression is stronger than that for treatment response. Sixteen of the registries collect data on disease progression. This allows patients to be tracked over time to determine how their disease changes whilst on treatment. A handful of studies were also found to be of potential value for determining treatment response and treatment effectiveness at various stages of the treatment sequence (ARTIS, NOR-DMARD, PsoBEST, ROB-FIN, SSATG). However, despite many of the registries collecting evidence over multiple lines of treatment, the lack of treatment response data (within 3 months) may limit the capacity to estimate the effect of subsequent lines of treatment.

Most of the registries identified were found to record details of patients withdrawing from treatment, including the exact date of withdrawal and the reason for withdrawal. Unfortunately, the usefulness of the registry evidence cannot be determined for estimating the effect of treatment withdrawal on disease progression, as this would rely on outcome measures (i.e. HAQ scores) being collected when patients withdraw from treatment. Only one registry—the HRBT—was explicitly identified as having done this. A number of registries also contain death data or are linked to a national death registry. These registries may potentially be useful for modeling the relationship between disease severity and mortality risk. With the exception of three registries, the degree of reporting on health care resource use in patients was poor. Two of the registries (ARAD and ARTIS) are linked to datasets containing health care utilization, and one registry (NOR-DMARD) contains visit data in addition to medications prescribed.

### Accessibility of PsA registries

Table 3 provides information pertaining to the accessibility of the registry data. The process for accessing registry data is vague for a number of the registries, and a number do not have any associated website or contact details. The requirement to pay an access fee may be a barrier for some analysts wanting to utilize data from the registries. A number of registries are not explicit about the financial requirements, whereas a small number explicitly state that an access fee will be charged (BSRBR, CORRONA and NOAR). This is usually tailored to the applicants’ status (clinical, non-clinical, student).

**Table 3:** Accessibility of PsA registries

	Application process	Financial require-ments	Website
ARAD	Application via website	–	https://arad.org.au/
ARTIS	–	–	–
ATTRA	–	–	http://attra.registry.cz/
BIOBADA-BRASIL	–	–	https://biobadaser.ser.es/biobadamerica/Brasil/index.html
BIOBADA-SER 2.0	–	–	https://biobadaser.ser.es
BSRBR	Application via website	Yes	http://www.rheumatology.org.uk/resources/bsr_biologics_registers/bsrbr_rheumatoid_arthritis_register
CORRONA	Enquiries via website	Yes	http://www.corrona.org/
DANBIO	Application via website	–	https://danbio-online.dk/
GISEA	–	–	http://www.gisea.eu/
HRBT	–	–	–
ICEBIO	–	–	–
NOAR	Application via website	Yes	http://www.uea.ac.uk/noar/home
NOR-DMARD	–	–	–
PsART	–	–	–
PsoBEST	–	–	https://www.psobest.de
Reuma	Application via website	–	www.reuma.pt
ROB-FIN	–	–	–
SCQM	Application via website	–	http://www.scqm.ch/
SSATG	–	–	–
SwePsA	–	–	–
UoT Psoriatic Arthritis	–	–	–

## Discussion

No individual registry on its own was found to address all of the questions set out in Box 1, and there was substantial variation regarding the extent to which the registries were useful for addressing each of the questions. Overall, the evidence pertaining to question 3 would appear to be strong given that the majority of the registries were found to collect the time-to-withdrawal from treatment in patients, as well as the reasons for withdrawal from treatment. Similarly, with regard to question 4, the majority of the registries collected at least one relevant outcome measure for assessing treatment effects. The fact that most of the registries follow patients over a long time horizon suggests that they would also be useful for estimating long-term disease progression (question 5).

The usefulness of the registry evidence could not be determined in many cases for the remaining research questions. With the exception of three registries, the degree of reporting on health care resource use in patients was poor (question 7). Likewise, there were only six registries reported as being potentially useful for modeling the relationship between disease severity and mortality risk (question 8). Finally, only one of the registries would appear to be capable of estimating the effect of treatment withdrawal on disease progression (question 6).

The review has also shown that there are notable disparities in the accessibility of the registries to researchers. Only seven of the registries identified had some form of formal application procedure in place for researchers to apply for data access, and of those registries, three had financial requirements. For nine of the remaining registries, the procedure for accessing data is complicated by the fact that there are no websites available to the public providing relevant contact details or information about the study protocol. Thus, the pool of registry evidence that we can say for certain that would be both accessible and useful for CEA research is limited, especially in the light of the financial barriers in place.

Another important consideration regarding the usefulness of registry evidence is the extent to which the data can be generalized across settings and jurisdictions. It is well established in the health economics literature that there are a number of key aspects related to a decision problem that may vary across jurisdictions [15–17]. These aspects include, but are not limited to, the appropriate health care interventions to be compared and the relevant patient subgroups to be investigated. Moreover, it is important to ensure that the evidence inputs selected are appropriate to the jurisdiction under investigation, e.g. appropriate health care unit costs and resource use estimates that correspond with the typical clinical practice. Consequently, these additional factors may further constrain the usefulness of the available registry data depending upon the specific decision problem under investigation.

In a similar manner, it is important to realize that current outcome tools, used for modeling disease progression, may be inadequate for this disease. The HAQ is a measure of function, mainly in the upper limb, and was developed for use in rheumatoid arthritis [18]. PsA is a complex heterogeneous disease which impacts joints in a less predictable manner—small joint involvement is less frequent, and lower limb joints may predominate over upper limb joints [19]. Moreover, there are other aspects of the condition that can impact upon function and quality of life such as enthesitis, dactylitis and spondylitis. Future studies that model outcomes should encompass these considerations. An outcome tool that measures across these domains, such as a generic quality of life outcome, is more appropriate for this disease.

### Limitations

It is important to acknowledge that the methodology used to identify previous or on-going registries in this study is subject to limitations. First, the definition of registry data employed in this study, taken from a report commissioned by the Agency for Healthcare Research and Quality, is a broad one that covers a range of different types of observational study. This definition presents an issue with regard to the practicality of identifying all potentially relevant registries given that it includes potentially huge numbers of small cohort studies. Related to the issue is the fact that, to the authors’ knowledge, there is no methodology available for the identification and selection of registry studies for CEA. This is largely driven by the lack of well-defined research question(s) in registry studies, especially when compared to the process of reviewing RCT evidence. This lack of research question is not conducive to a search-strategy based on keyword terms. Instead, the authors decided that a pearl growing approach would offer greater flexibility. It is important to emphasize that there may be additional benefits associated with registry evidence beyond those considered in this paper. For instance, registry data can circumvent some of the problems relating to the generalizability of evidence from clinical trials, which can occur as a result of strict inclusion criteria, by recruiting more inclusive populations of patients [20].

### Lessons for the future

There are a number of recommendations regarding the future collection of data in PsA registries that can be made based upon the findings in this review. Firstly, the timing of the data collection in many of the registries was incompatible with the requirements of the modeling techniques used in contemporary CEA studies [11]. For future research, we recommend that data collection should take place 3 months after the initiation of a treatment in PsA patients. In addition, the collection of outcome measures in patients when they withdraw from treatment would permit researchers to obtain empirically derived estimates of disease progression, rather than having to rely upon assumptions.

A second issue relates to the selection of variables. Only a small number of registries are currently collecting the optimal outcomes for estimating the initial response to treatment. To this end, future data collection should ideally include PsARC, PASI75 and HAQ outcome measures. Furthermore, there were very few cases where registries could be identified as having collected information relating to patient mortality or health care resource use. Data collection pertaining to each of these facets would greatly enhance the robustness of the evidence used in future studies investigating the cost-effectiveness of PsA treatments.

Finally, with regard to the generalizability of evidence across jurisdictions, there are lessons that can be learned from data registries established in other disease areas. For example, the MDS-RIGHT database was established to monitor outcomes in patients with myelodysplastic syndromes across 17 countries using the same study protocol [21]. The advantage of this approach is that it allows researchers to account for variations occurring in clinical practice across countries.

## Conclusions

In recent years, there has been increased enthusiasm amongst the health research community around the opportunities that may be afforded by registry data for the evaluation of health care interventions [22, 23]. The objective of this paper was to understand these opportunities in the context of cost-effectiveness modeling for PsA treatments. A review of the literature identified 21 potentially relevant registries from around the world containing patients with PsA. Most of the registries identified were shown to be at least partly useful in informing the evidence requirements as specified in previous modeling efforts in this area. Overall, however, the registries were generally lacking in evidence pertaining to the estimation of initial treatment responses, disease progression following withdrawal from treatment and the optimum sequences of treatments for PsA. Moreover, there were a number of cases in which the review was unable to determine the usefulness of the registry data; namely, the collection of information about health care resource use and patient mortality, as well as data accessibility. It is hoped that the findings of this study will provide the research community with a greater understanding of the current opportunities available with regard to the application of existing PsA registries for CEA. Furthermore, it is hoped that the recommendations provided will inform future data collection within a registry setting.
